# Therapeutic Hypothermia in Spinal Cord Injury: The Status of Its Use and Open Questions

**DOI:** 10.3390/ijms160816848

**Published:** 2015-07-24

**Authors:** Jiaqiong Wang, Damien D. Pearse

**Affiliations:** 1 The Miami Project to Cure Paralysis, University of Miami Miller School of Medicine, the Lois Pope Life Center, Locator code (R-48), PO BOX 016960, Miami, FL 33136, USA; 2 The Department of Neurological Surgery, University of Miami Miller School of Medicine, the Lois Pope Life Center, Locator code (R-48), PO BOX 016960, Miami, FL 33136, USA; 3 The Neuroscience Program, University of Miami Miller School of Medicine, the Lois Pope Life Center, Locator code (R-48), PO BOX 016960, Miami, FL 33136, USA; 4 The Interdisciplinary Stem Cell Institute, University of Miami Miller School of Medicine, the Lois Pope Life Center, Locator code (R-48), PO BOX 016960, Miami, FL 33136, USA

**Keywords:** hypothermia, spinal cord injury, neuroprotectivion, cell death, inflammation, cooling, transplantation, angiogenesis, free radicals

## Abstract

Spinal cord injury (SCI) is a major health problem and is associated with a diversity of neurological symptoms. Pathophysiologically, dysfunction after SCI results from the culmination of tissue damage produced both by the primary insult and a range of secondary injury mechanisms. The application of hypothermia has been demonstrated to be neuroprotective after SCI in both experimental and human studies. The myriad of protective mechanisms of hypothermia include the slowing down of metabolism, decreasing free radical generation, inhibiting excitotoxicity and apoptosis, ameliorating inflammation, preserving the blood spinal cord barrier, inhibiting astrogliosis, promoting angiogenesis, as well as decreasing axonal damage and encouraging neurogenesis. Hypothermia has also been combined with other interventions, such as antioxidants, anesthetics, alkalinization and cell transplantation for additional benefit. Although a large body of work has reported on the effectiveness of hypothermia as a neuroprotective approach after SCI and its application has been translated to the clinic, a number of questions still remain regarding its use, including the identification of hypothermia’s therapeutic window, optimal duration and the most appropriate rewarming rate. In addition, it is necessary to investigate the neuroprotective effect of combining therapeutic hypothermia with other treatment strategies for putative synergies, particularly those involving neurorepair.

## 1. Spinal Cord Injury (SCI) Epidemiology and Pathophysiology

Spinal cord injury (SCI) is a major health problem around the world. In the United States, it is estimated that 12,000 to 20,000 new SCIs occur each year, and currently over 200,000 Americans are living with SCI [1]. The most common causes of SCI are motor vehicle accidents, falls and sports injuries. For people younger than 65, the leading cause of SCI is motor vehicle accidents. Among people older than 65, SCI is mostly induced by falls [1]. SCI often results in a diverse range of neurological symptoms that depend on the severity and specific spinal cord level of the injury. Cervical SCI often results in tetraplegia, difficulty breathing, and dysregulation of autonomic functions including heart rate, blood pressure and body temperature. Thoracic and lumbosacral SCI often produces locomotor dysfunction in the legs and hips, the loss of control of the bowel and bladder, as well as sexual dysfunction.

Pathophysiologically, tissue damage after SCI is divided into two separate phases; the primary injury and the secondary injury. The primary injury occurs at the moment of SCI impact. It is commonly related to spinal cord compression/transection due to a vertebral bone fracture or distraction/stretching of the spinal column [2,3,4]. The secondary mechanisms of SCI are composed of a cascade of temporal events that include neurogenic shock [5,6], hemorrhage and ischemia-reperfusion [7,8], an increase of intracellular calcium and calcium-mediated activation of proteases (e.g., calpain) and lipases [9,10,11,12,13], mitochondrion dysfunction, the generation of free radicals and nitric oxide as well as other oxidants [14], the release of excitatory neurotransmitters (e.g., glutamate) and excitotoxicity [15,16], which in turn results in the apoptosis of neurons, oligodendrocytes, microglia and astrocytes [17,18,19], Wallerian degeneration of injured axons [20], the infiltration of neutrophils and the migration of macrophages and microglia [21,22,23,24] as well as astrocyte activation and scar formation [25]. These secondary pathomechanisms are associated with the majority of the morbidity and mortality after SCI.

## 2. Treating SCI and the Application of Hypothermia

To date, the treatments for SCI have been limited to methylprednisolone, surgical interventions and rehabilitation. However, these clinical approaches are not sufficient to provide significant recovery of function after SCI to the majority of individuals. Accordingly, it is necessary to develop novel therapeutic interventions to delay the progression of the pathophysiological processes associated with secondary injury to limit the degree of SCI-induced neurological dysfunction, such as hypothermia. Previously, the therapeutic use of hypothermia has been investigated clinically in cardiac arrest [26,27], neonatal hypoxic ischemic encephalopathy [28,29], hepatic encephalopathy [30,31], aneurysmal brain surgery [32], hemorrhagic stroke [33,34], traumatic brain injury [35,36,37,38] and SCI [39,40,41,42].

Based upon the level of temperature reduction, hypothermia can be divided into three levels: profound hypothermia (less than 30 °C), moderate hypothermia (30–32 °C) and modest hypothermia (32–34 °C). Early hypothermia studies on the surgical treatment of aneurysms of the transverse aortic arch compared the mortality between hypothermia administered at 12–16 or 24–26 °C. It was reported that the 12–16 °C hypothermia group had 50% mortality, as opposed to 20% mortality in the 24–26 °C hypothermia group [43]. In an *in vitro* murine spinal cord culture model, Lucas [44] observed that when the temperature was below 17 °C, the neuronal perikarya and dendrites swelled, with the majority of the swollen neurons dying during the phase of rewarming to 37 °C. Most recently, it has been reported in a piglet hypoxia-ischemia model that mild (35 °C) and moderate (33.5 °C) whole body cooling reduced brain cell death and microglia activation, compared to overcooling (30 °C), which was associated with detrimental pathological effects within some brain regions, such as the mid-temporal cortex, periventricular white matter, caudate, putamen and thalamus [45]. These studies have suggested that the temperature of hypothermia should be limited to the mild to moderate range so as to obviate the adverse effects associated with low temperatures and/or the transition back to regular body temperature. Therefore in recent investigations, modest hypothermia (32–34 °C) has been the preferred target for cooling strategies to provide neuroprotection.

## 3. The Experimental and Clinical Application of Hypothermia in SCI

To date, the neuroprotective effect of hypothermia has been demonstrated in both experimental and human SCI studies.

### 3.1. Hypothermia in Experimental SCI

Hypothermia has been investigated in a wide diversity of animal SCI models. The earliest hypothermia studies were focused on local hypothermia, in which neurological benefit was observed in dogs, monkeys and pigs after SCI [46,47,48,49,50]. However, compared to local hypothermia, a superior neurological protective effect was observed when hypothermia was applied systemically in animal SCI models utilizing rabbits, pigs, dogs and rats [51,52,53,54]. In a rat thoracic spinal cord contusion model, Yu and colleagues [54] applied modest systemic hypothermia (32–33 °C) at 30 min post-injury for a period of 4 h. This application of modest systemic hypothermia improved locomotor function as demonstrated by higher Basso-Beattie-Bresnahan (BBB) locomotor scores as well as a significant reduction in the area of tissue loss at both seven days and 44 days post-injury [54].

**Figure 1:**
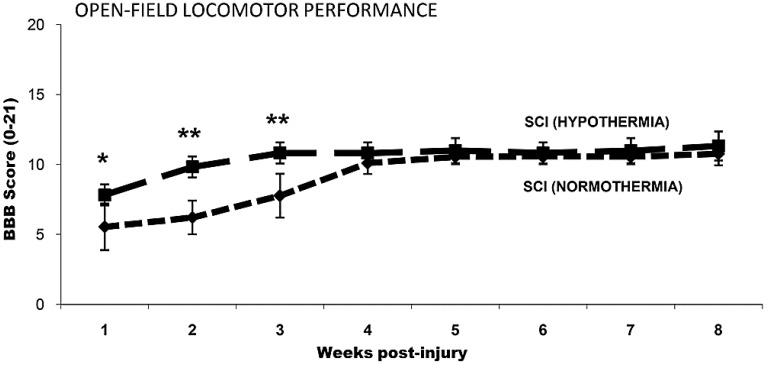
Open field locomotor ability was significantly improved acutely but not persistently following hypothermia. Locomotor performance was assessed from one week post-injury through eight weeks according to the Basso-Beattie-Bresnahan (BBB) scale. Although a significant increase in BBB scores was observed transiently one to three weeks after injury with hypothermia over normothermic controls, both groups achieved similar behavioral endpoints by eight weeks. Data are expressed as average ± standard error of the mean. Group numbers: *n* = 9 normothermic injured group and *n* = 9 hypothermic injured group. ** *p* < 0.01, * *p* < 0.05 compared with normothermic controls. Reprinted with permission from [42], copyright Wiley-Liss, Inc., 2009.

**Figure 2:**
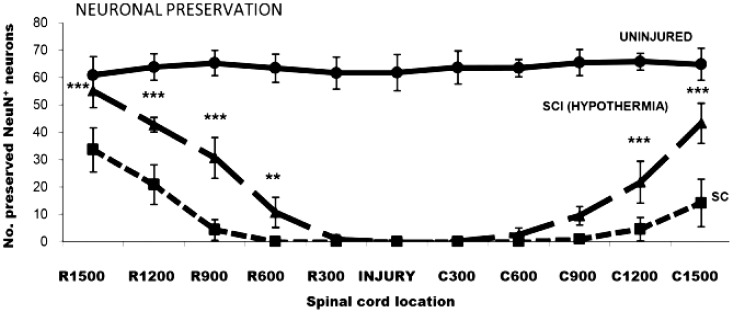
Hypothermia increased the numbers of preserved ventral motor neurons rostral and caudal to the injury site at 10 weeks post cervical spinal cord injury (SCI). Counts of cells labeled for NeuN (a neuron-specific marker) from transverse sections rostral (R) and caudal (C) to and within the injury epicenter of the cervical cord showed that acute application of mild systemic hypothermia could significantly increase the numbers of NeuN-immunoreactive neurons in the ventral horn (laminae VII–IX) at distances of 900 µm and greater from the injury epicenter compared with normothermic controls. Almost no preserved ventral motor neurons, however, were detected within the immediate injury site in both SCI groups. Recordings from uninjured controls are provided for comparison, and the data are expressed as the average ± standard error of the mean. *** *p* < 0.001, ** *p* < 0.01 compared with normothermic controls. Reprinted with permission from [42], copyright Wiley-Liss, Inc., 2009.

**Figure 3:**
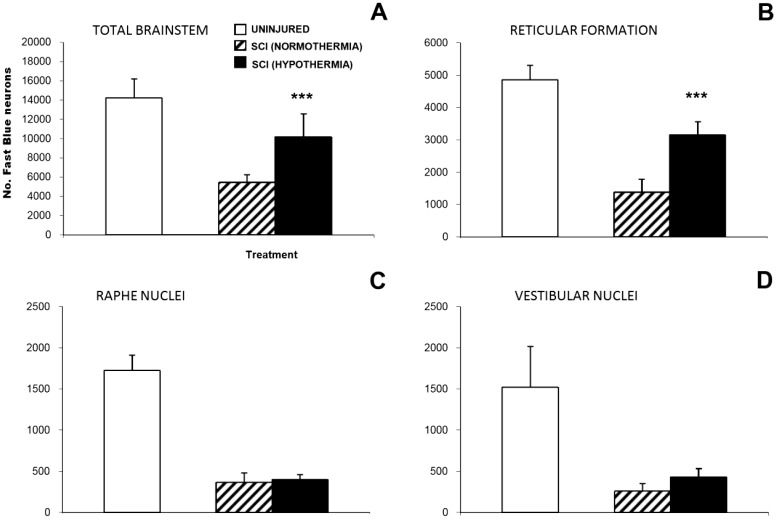
Retrograde tracing analysis reveals that acute hypothermia resulted in greater sparing of brainstem axons projecting caudal to the injury site at 10 weeks post cervical spinal cord injury. (**A**) Retrograde labeling of neuronal somata with fast blue (FB), provided caudal to the lesion, shows that there was a greater sparing of brainstem projections (indicated by an increase in numbers of labeled neuronal perikarya) when acute hypothermia was applied. Investigation of specific brainstem neuronal populations revealed a significant increase in retrogradely labeled neurons in the reticular formation (**B**) but not the raphe (**C**) or vestibular nuclei (**D**) after hypothermia treatment. Recordings from uninjured controls are provided for comparison, and the data are expressed as the average ± standard error of the mean. *** *p* < 0.001 compared with normothermic controls. Reprinted with permission from [42], copyright Wiley-Liss, Inc., 2009.

Previously, our laboratory utilized a rat cervical spinal cord contusion injury model, with the induction of mild systemic hypothermia (33 °C) beginning within 5 min of injury for a duration of 4 h, with slow rewarming at the rate of 1 °C per hour [42]. This acute, mild hypothermia paradigm resulted in an improvement in limb function as demonstrated by significantly greater upper body strength and a faster recovery in BBB scores during the one to three weeks post-cervical contusion period (Figure 1) [42]. Additionally, histological evaluation for the injured spinal cord segment from rats that survived 10 weeks after cervical SCI revealed a histological improvement with increased preservation of both white matter and gray matter, as well as higher numbers of surviving ventral motor neurons (Figure 2) [42] and intact axons (Figure 3) [42]. Batchelor and colleagues [55] utilized a rat spinal cord compression model to show that mild systemic hypothermia (33 °C) could rapidly decrease intracanal pressure, indicating that hypothermia may be a useful approach to acutely decompress the spinal cord before surgical decompression. In addition, Maybhate and colleagues [56] applied transient systemic hypothermia (32 ± 0.5 °C) for 2 h beginning at 2 h post-injury in a rat thoracic spinal cord contusion model. They observed that early systemic hypothermia provided significant neuroprotection as demonstrated (1) electrophysiologically, the hypothermia group presented higher amplitudes in somatosensory evoked potentials; (2) functionally, the hypothermia group showed much better BBB scores immediately after injury and at four weeks post-injury; and (3) histologically, more tissue was maintained in the hypothermia group.

Most recently, Saito and colleagues [57] have shown in a spinal cord ischemia model that very mild hypothermia of only a 1 °C decrease in body temperature to 36.3 °C, resulted in greater preservation of motor neurons, decreased white matter vacuolation, reduced astrogliosis in both white matter and gray matter, and led to a concomitant improvement in locomotor function. Similarly, Grulova and colleagues [41] have shown that systemic hypothermia of 32.0 °C, could promote neuronal survival and improve locomotor function, as well as urinary bladder activity in a rat thoracic spinal cord compression model.

All of these animal experiments have provided support for the beneficial neuroprotective effects of mild and moderate hypothermia after SCI and set the stage for translating this therapeutic approach into the clinic for SCI management.

### 3.2. Hypothermia in Human SCI

In order to translate hypothermia from animal experimentation into the clinic, it is also necessary to test the safety and efficacy of this approach in small cohorts of SCI patients. The first trial of hypothermia on human beings was performed in a clinical case study involving a patient with an extensive spinal epidural abscess [58]. Later, Demian and colleagues [59] reported that localized spinal cord cooling resulted in an impressively beneficial effect for three acute cervical SCI patients, without a significant change in whole body temperature, vascular dynamics or respiration parameters. Later, more patients were tested with local hypothermia via irrigating the subdural space with cold saline at 5 °C for about 2 h [60,61]. Romodanov and colleagues [62] performed a spinal cord hypothermia study on 113 patients and reported that hypothermia decreased spinal cord bleeding and edema, reduced muscle spasticity, improved the motor function of the affected limbs, and produced an analgesic effect. Similar beneficial effects were observed when local hypothermia was performed on the spinal cord in the postoperative period [63]. Cherkashina [64] stated that the beneficial effect of local hypothermia was due to a decreased excitability of spinal cord alpha motoneurons, increased local blood flow in the spinal cord and a raised vascular tension. It has been demonstrated that hypothermic circulatory arrest can effectively prevent the development of SCI associated with extensive aortic resection [65] and decrease the incidence of neurologic deficits after thoracoabdominal aneurysm repair [66,67,68].

**Figure 4:**
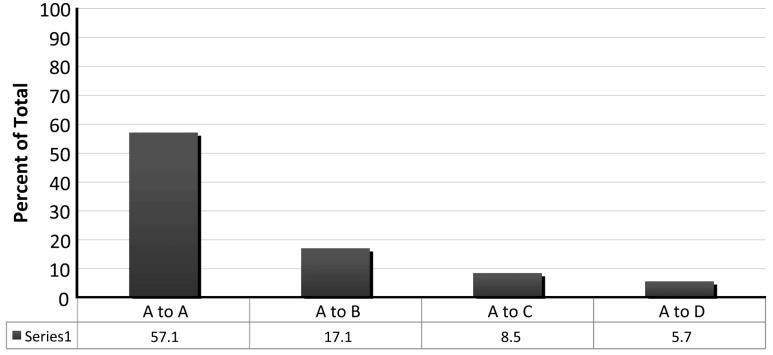
ISNCSCI outcome in 31 patients with initial and stable ISNCSCI A who did not improve within first 24 h from admission. Reprinted with permission from [40], Nature Publishing Group, 2013.

Following, and with continued testing of local hypothermia, systemic hypothermia moved from experimental studies to the clinic. Hayes and colleagues [69] used the circulation of propylene glycol through a “microclimate” head and vest garment on patients with chronic compressive or contusive SCI to induce mild hypothermia (−1 °C). For some SCI patients, this systemic mild cooling approach increased the amplitude of cortical somatosensory evoked potentials, suggesting that cooling could improve central conduction in some SCI patients with conduction deficits [69]. The most recent published studies examining systemic hypothermia as a treatment for human SCI have come from The Miami Project to Cure Paralysis, University of Miami Miller School of Medicine [39,40,70,71,72,73,74,75]. Levi and colleagues [73] reported that patients with acute cervical SCI could be cooled relatively safely using endovascular catheters and that there was an excellent correlation between intravascular temperature and intrathecal cerebrospinal fluid temperature. This technique cooled the patient to the target temperature (33 °C) in 2.72 ± 0.42 h, and could be maintained for 48 h without increasing the risk of cardiac arrhythmias, deep vein thrombosis or pneumonia [73]. Cappuccino and colleagues [70] reported a case study of a National Football League player who suffered from a complete cervical SCI resulting from a C3/4 fracture dislocation with complete motor paralysis and sensory loss (American Spinal Injury Association (ASIA) Impairment Scale A). This patient received moderate systemic hypothermia (33.5 °C) immediately after SCI, in addition to traditional surgical decompression and methylprednisolone. After 36 h of hypothermia, a rapid neurologic improvement was observed in the individual, and with rehabilitation, there was an overall ASIA conversion from A to D [70]. In another study involving a small group of five iatrogenic SCI patients, Madhavan and colleagues [74] carried out moderate systemic hypothermia (33 °C) immediately post-surgery with maintenance for 24 h postoperatively. Patients were rewarmed at a rate of 0.1 °C per hour and four were also administered methylprednisolone. This management paradigm showed patient ASIA score improvements of 1–2 grades [74]. Recently, Dididze and colleagues [40] reported on the results of a case-controlled study comprising thirty-five acute cervical SCI patients who, on admission, were AISA A. These individuals received modest, intravascular hypothermia (33 °C) via a femoral vein catheter beginning at 5.76 ± 0.45 h post-injury (four cases with delayed admission were excluded). The body temperature was maintained at 33 °C for 48 h, followed by slow rewarming at the rate of 0.1 °C per hour (taking about 24 h to return to 37 °C). In this study, four patients converted from International Standards for Neurological Classification of Spinal Cord Injury scale (ISNCSCI) A to ISNCSCI B within the first 24 h. In the other 31 patients with consistent ISNCSCI A during the first 24 h, 35.5% of the patients (11/31) showed an increase of at least one grade in ISNCSCI at latest follow up, 10.07 ± 1.03 months (Figure 4) [40]. Among those four patients who improved from ISNCSCI A to ISNCSCI B within the first 24 h, one increased to ISNCSCI C, two upgraded to ISNCSCI D and one converted to ISNCSCI E [40]. Respiratory complications were similar among retrospective and prospective groups, and the thromboembolic complication rate was 14.2% [40]. All these preliminary human studies showed evidence of the beneficial therapeutic effect of systemic hypothermia in acute SCI. Due to the limited number of patients involved in these studies, however, a multi-center, randomized and blinded study is required to evaluate whether systemic hypothermia is appropriate for the treatment for acute SCI.

## 4. Mechanisms of Hypothermia-Mediated Protection

As described above, hypothermia has provided a significant benefit in both experimental and human SCI. The neuroprotective action of hypothermia has been ascribed to its effect on a number of pathomechanisms including, the slowing of metabolism, decreasing cellular stress and reducing the generation of free radicals, ameliorating inflammation and inhibiting excitotoxicity that can reduce apoptosis, preserving the blood spinal cord barrier, preventing vasogenic edema, inhibiting astrogliosis, decreasing axonal damage, promoting neurogenesis as well as increasing angiogenesis [76,77,78].

### 4.1. Slowing down the Rate of Metabolism and Decreasing the Generation of Free Radicals

Hypothermia lowers the metabolic rate of neurons after SCI, inhibiting the generation of lactate, hydrogen and phosphate, as well as preserving glucose. In a dog thoracoabdominal aortic clamping model, Dzsinich and colleagues [79] showed that levels of glucose, lactate, pCO_2_ and Neuron Specific Enolase (NSE) in the cerebrospinal fluid (CSF) was significantly altered. Regional spinal cord hypothermia with icy peridural irrigation reduced the presence of anaerobe metabolites found in the CSF [79]. Similarly, in a rabbit spinal cord ischemia and reperfusion model, Allen and colleagues [80] reported that regional spinal cord hypothermia with cold heparinized saline perfusion could inhibit decreases in adenosine triphosphate and glucose concentrations, while preserving intracellular concentrations of glutamate and aspartate, to prevent the incidence of paraplegia. Further, in humans with a spinal cord ischemic injury, a study demonstrated that the combination of a distal femoral bypass and hypothermia (30 °C) reduced the lactate concentration in CSF [81]. All these findings are consistent with the effect of hypothermia on attenuating neuronal metabolic rate.

In addition to slowing down neuronal metabolism, hypothermia also decreases the generation of free radicals, such as super oxide, nitric oxide and hydroxal radicals. As described previously, the generation of free radicals is a secondary pathological mechanism of SCI [14]. In an *in vitro* guinea pig spinal cord compression injury model, the amount of reactive oxygen species increased dramatically within 5 s following compression of the spinal cord [82]. Hypothermia, however, significantly inhibited superoxide and lipid peroxidation [82,83]. The tissue content of malonildialdehyde (MDA) is a reliable parameter for measuring lipid peroxidation. In a rat spinal cord compression model, Tüzgen and colleagues [83] utilized chilled normal saline solution to induce epidural cooling, and demonstrated that the tissue MDA levels decreased significantly after application. These findings suggested that hypothermia could play a significant role in inhibiting lipid membrane peroxidation and the subsequent generation of free radicals.

### 4.2. Inhibiting Excitotoxicity and Neural Cell Apoptosis

Hypothermia also provides a neuroprotective benefit by decreasing the release of excitatory neurotransmitters and ensuing excitotoxicity. Glutamate excitotoxicity plays an important role in the pathogenesis of SCI [2,84]. Excessive release of glutamate and excitotoxicity is a major pathological event in cellular damage after acute SCI [85]. In a rat spinal cord ischemia model, Ishikawa and Marsala [86] reported that mild hypothermia (33 °C) prevented the release of glutamate during ischemia and after reperfusion. Similarly, in a rabbit spinal cord ischemia model, Wakamatsu and colleagues [87] observed that the concentration of glutamate was decreased after moderate hypothermia (32 °C). Alternatively to *in vivo* paradigms, Nishi and colleagues [88] performed whole-cell patch-clamp recordings in a spinal cord slice ischemia model, and observed that excitatory synaptic transmission was significantly suppressed by hypothermia when given from 28 to 32 °C and when further decreased to 24 °C, resulting in reduced neuronal death. All these findings from *in vivo* and *in vitro* experiments are consistent with the hypothermia’s beneficial mechanism in inhibiting the release of excitatory neurotransmitters and subsequent excitotoxicity.

In addition to decreasing excitotoxicity, hypothermia also works by antagonizing the apoptotic signaling machinery within neurons and glia following SCI. Neuronal and glial apoptosis and death has been demonstrated as a prominent feature after SCI. Specifically after SCI, an increase in the expression of apoptotic molecules p53 and Bax, together with the activation of caspase-3 and caspase 8, the cysteine proteases that execute the apoptotic cell death program, has been observed [89,90,91,92]. In a rat spinal cord contusion injury model, Ok and colleagues [93] reported that both moderate, epidural hypothermia (28 °C for 48 h) and moderate systemic hypothermia (32 °C for 48 h) significantly decreased the expression of caspase-8 and caspase-9, while moderate systemic hypothermia additionally reduced the expression of caspase-3. On the other hand, Wang and colleagues [94] showed that hypothermia could also retard neuron apoptosis by increasing the expression of anti-apoptotic bcl-2 and inhibiting the expression of p53 in a rabbit spinal cord ischemia model. All these experimental findings suggest that hypothermia could inhibit apoptosis in injured spinal cord.

### 4.3. Ameliorating Inflammation

Along with the prevention of apoptosis, inflammation must also be controlled. Hypothermia produces a profound dampening of the inflammatory response. Microglia/macrophage activation is a common secondary pathological process following SCI [95,96]. It has been demonstrated that the infiltration of microglia/macrophages at the site of SCI and their contact with dystrophic axons can result in extensive axon retraction [22,97]. In a rat spinal cord contusion model, Ok and colleagues [92] reported that after treatment with either moderate epidural hypothermia (28 °C for 48 h) or moderate systemic hypothermia (32 °C for 48 h), numbers of OX-42 positive cells (microglia/macrophages) were significantly decreased, as was the expression of the p38 mitogen-activated protein kinases, which are involved in immune cell activation [98,99]. Morino and colleagues [78], utilizing a mild spinal cord compression model, revealed that mild hypothermia (33 °C for 1 h) inhibited the proliferation of microglia and decreased levels of tumor necrosis factor-alpha (TNF-α). This was in turn associated with an improvement in motor function. To measure the effect of hypothermia on neutrophil activity, Chatzipanteli and colleagues [76] utilized a T10 thoracic spinal cord contusion model. They observed that myeloperoxidase (MPO) activity (a marker of neutrophil accumulation) was elevated at 3 and 24 h post-injury in the normothermia group. MPO activity in the hypothermia group (32.4 °C for 3 h) was significantly decreased at 24 h post-injury within the injured spinal cord segment [76]. All these reports suggest the role hypothermia can play in ameliorating the inflammatory response after SCI.

### 4.4. Preserving the Blood Spinal Cord Barrier and Preventing Edema

Hypothermia can also provide a neuroprotective effect through its ability to preserve the blood-spinal cord barrier (BSCB) and ameliorate local edema. Normally, the BSCB regulates the fluid microenvironment within the spinal cord [100]. SCI results in the disruption of the BSCB, increasing its permeability to proteins and molecules, and producing edema [101,102]. Yu and colleagues [103] utilizing a T8-9 spinal cord compression model, showed that moderate, systemic hypothermia (30 °C), via wetting the animals with a 20% ethanol/water solution at room temperature (20 °C), decreased the extravasation of the plasma proteins albumin, fibrinogen and fibronectin. In a clinical study involving 61 spinalized patients treated during the postoperative period, Iumashev and colleagues [63] demonstrated that local spinal cord application of hypothermia prevented edema formation. Similarly, a reduction in spinal cord edema was also reported in a study involving spinal cord hypothermia conducted in 113 patients [62]. Therefore, both experimental and human studies suggest that hypothermia could provide protective effects via the preservation of the BSCB.

### 4.5. Inhibiting Astrogliosis and Increasing Angiogenesis

Another beneficial mechanism of hypothermia is to retard astrogliosis and scar formation. Reactive astrogliosis is a hallmark feature of SCI, taking place at the lesion site days to weeks after injury and providing a major impediment to endogenous axon growth [104,105]. The application of hypothermia has been shown to inhibit astrogliosis. In a rat spinal cord ischemia model, Saito and colleagues [57] reported that the percent staining area positive for glial fibrillary acidic protein (GFAP) was decreased in both gray and white matter after mild hypothermia (1 °C decrease in body temperature to 36.3 °C, initiated 15 min prior to ischemia and maintained during ischemia).

In addition to inhibiting astrogliosis, it has been reported that hypothermia can promote the expression of pro-angiogenic factors. Vascular disruption is a common traumatic event after SCI, resulting in hemorrhage, a reduction in spinal cord blood flow and ischemia [106,107,108]. Accordingly, inducing angiogenesis is a therapeutic strategy to promote spinal cord repair. In a rat compression SCI model, Kao and colleagues [109] applied systemic hypothermia (33 °C), and observed an increase in the expression of vascular endothelial growth factors and the number of bromodeoxyuridine-positive endothelial cells, suggesting that hypothermia can exert a beneficial effect by promoting angiogenesis.

### 4.6. Decreasing Axonal Damage and Promoting Neurogenesis

Hypothermia can also provide a protective benefit through reducing axonal damage after SCI. Axonal injury is characterized by axonal cytoskeletal disorganization, impaired axonal transport as well as axonal swelling, disconnection, dieback and degeneration [110,111,112]. In a rat spinal cord compression model, Westergren and colleagues [113] performed systemic hypothermia and reduced the core temperature from 38 to 30 °C. The number of abnormal axons as identified by the accumulation of beta-APP, ubiquitin and PGP-9.5, were much lower in the peri-injury zone after hypothermia treatment, indicating an axonal protective effect of hypothermia [113].

Hypothermia may also influence neurogenesis after SCI. Neural cell death is a common pathological process following SCI, characterized by apoptosis and/or necrosis [114]. Neurogenesis provides the potential to produce new cells, replacing the dead neural cells and restoring function. In a rat spinal cord compression model, Kao and colleagues [109] showed that systemic hypothermia (33 °C) produced an increase in the numbers of both glial cell line-derived neurotrophic growth factors and bromodeoxyuridine-neuronal-specific nuclear protein double positive cells within the injured spinal cord at four days after SCI.

In summary, hypothermia has shown protective effects after SCI through a variety of mechanisms, including the slowing of neuronal metabolic rate, the inhibition of free radical production, a decrease in excitotoxicity, inflammation, apoptosis and astrogliosis, a preservation of blood-spinal cord-barrier and axonal protection as well as providing a foundation for neurorepair through promoting angiogenesis and neurogenesis.

## 5. The Combined Use of Hypothermia with Other Therapeutic Approaches

Due to the known mechanisms of hypothermia’s effects after SCI, researchers have taken an interest in combining hypothermia with other therapeutic approaches that would provide synergistic benefit. These approaches include combining hypothermia with free radical scavengers, hyperbaric oxygen, alkalinization, local anesthesia, antioxidants and cell transplantation.

### 5.1. Hypothermia and Antioxidants

As described previously, the increased generation of free radicals plays an important role in the pathogenesis of SCI. Hypothermia alone has been demonstrated to decrease the generation of free radicals, such as super oxide, nitric oxide and hydroxyl radicals. Recent studies have reported that the combination of hypothermia with radical scavengers or antioxidants, can provide beneficial synergistic effects after SCI. For example, while the injection of the radical scavenger edaravone or the application of topical cooling with transvertebral cooling pads have each been demonstrated to decrease spinal cord damage, their combination provides a greater protective effect [115]. Another antioxidant approach is the use of hyperbaric oxygen. Topuz [116] reported that the combination of hypothermia and hyperbaric oxygen prevented the elevation of MDA levels in spinal cord tissue, and increased the activity of antioxidant enzymes, such as superoxide dismutase (SOD), glutathione peroxidase (GSH-Px) and catalase (CAT). The combination of hypothermia and hyperbaric oxygen was shown to be superior to either approach alone, indicating their synergistic benefits in ameliorating spinal cord secondary damage [115]. In addition to hyperbaric oxygen, administration of *N*-acetylcysteine (NAC) has also been employed with hypothermia. Previously, NAC has been primarily used in the treatment of acetaminophen poisoning and chronic bronchitis [117,118]. Cuzzocrea and colleagues [119] demonstrated that NAC could improve neuronal survival in the hippocampus of Mongolian gerbils subjected to transient cerebral ischaemia. Cakir and colleagues [120] later combined NAC with hypothermia (34–35 °C) to treat rabbits exposed to spinal cord ischemia-reperfusion injury, discovering that this combination could provide significantly greater recovery of motor function, with accompanying preservation of neurons and minimal immune cell infiltration.

### 5.2. Hypothermia and Alkalinization

Another combinational approach that has been evaluated with hypothermia is alkalinization. SCI is associated with the release of excitatory amino acids such as glutamate and aspartate, which acidify the extracellular environment [121,122]. This decrease in pH within the microenvironment subsequently facilitates the influx of calcium ions into neurons and induces neurotoxicity [123]. Therefore, alkalinization is able to block calcium-induced neurotoxicity and provide neuroprotection [124,125]. Recently, Kuffler [126,127] compared the neuroprotective effects of hypothermia and alkalinization, either alone or in combination, in an adult human dorsal root ganglion ischemia model. It was demonstrated that when applied individually, hypothermia provided a four-fold increase in neuroprotection and alkalinization provided an eight-fold increase. However, their combination enhanced neuroprotection by 26-fold. These data suggest that neuroprotection could be greatly augmented when hypothermia was combined with alkalinization.

### 5.3. Hypothermia and Anesthetics

Combining hypothermia with anesthetics has also shown promise. Recently anesthetics have been evaluated to determine whether they can ameliorate pathological changes after SCI [128]. For example, ketamine has been demonstrated to be neuroprotective after SCI in rats through antagonizing inflammation (decreasing TNF-α and IL-6), reducing oxygen free radicals (lowered MDA levels) and anti-apoptotic effects (fewer TUNEL-positive cells) [128]. Another anesthetic, Bupivacaine, works by blocking potassium conduction, reducing neuronal depolarization and preserving glucose and oxygen to help maintain normal membrane potentials [129]. In a rat transient spinal cord ischemia model, Lee and colleagues [130] showed that when intrathecal bupivacaine (0.5%) was accompanied by hypothermia, motor and sensory deficit scores were significantly less than when either single treatment was used. A similar synergistic effect after SCI in the rat was observed when the delta-opioid agonist SNC80 was administered intrathecally under mildly hypothermic (35 °C) conditions [131]. These findings indicate that the combination of anesthetics with hypothermia can provide enhanced neuroprotection after SCI.

### 5.4. Hypothermia and Cell Transplantation

While the majority of combination approaches used with hypothermia have focused on enhancing neuroprotection, cell transplantation offers a strategy that can provide added benefit through the complementary mechanism of neurorepair. In a rat SCI model, Wang and colleagues [104] combined the transplantation of bone marrow mesenchymal stem cells with mild hypothermia (33–35 °C) and reported a synergistic effect. Histologically, animals in this combined treatment group showed no cavity formation and a significantly greater axonal preservation than the animals receiving only bone marrow mesenchymal stem cell transplantation [104]. Functionally, BBB scores after SCI showed improved locomotor recovery when mild hypothermia was combined with bone marrow mesenchymal stem cell transplantation than when bone marrow mesenchymal stem cell transplantation was used alone [104]. In another study by Wang and Zhang [132], neural stem cell (NSC) transplantation was combined with mild systemic hypothermia (34 ± 0.5 °C) in a rat spinal cord hemisection model. Hind limb motor function was found to be superior in the combined treatment paradigm compared to those animals that only received NSC transplantation. Histologically, more axonal-like structures were observed in the lesion with the combined treatment of NSC transplantation and hypothermia, compared to NSC transplantation alone. The enhanced neuroprotective effect of such a combinational treatment may be due to the fact that hypothermia optimizes the microenvironment of the injured spinal cord for repair, and supports the survival of the implanted cells.

### 5.5. Other Potential Combinatory Approaches that May Provide Synergistic Benefit with Hypothermia after SCI

In addition to the above combinatory approaches, many other promising medications and cell therapies could be utilized in combination with hypothermia for SCI repair. These therapies include lead clinical candidates erythropoietin, riluzole, the calpain inhibitor AK295 [133], minocycline [134], the Rho inhibitor Fasudil [135], and Schwann cell (SC) transplantation. The evaluation of these putative combinations in experimental SCI paradigms may pave the way for more effective therapies that can be readily moved into clinical investigations.

## 6. Currently Unanswered Questions Regarding the Optimal Use of Hypothermia in SCI

Aside from exploring novel combinational approaches with hypothermia for enhanced therapeutic effect, a number of questions remain regarding optimal hypothermia application after SCI. These include the relationship between the therapeutic window and duration of hypothermia as well the rate of rewarming to normothermia so as to optimize its therapeutic potential.

### 6.1. Therapeutic Window

The time window during which cooling should be started after SCI has not been well standardized in both experimental and human studies. Albin [47] and White [136], in a monkey spinal cord weight drop SCI model, considered that a 4 h period after injury was the critical time window in which cooling needed to be initiated. Once initiated, hypothermia should then be maintained for a minimum of 3 h to provide protection and functional benefit. During the recent decade of experimental SCI research, the majority of studies have targeted the use of systemic hypothermia immediately post-injury [41,42,55,56,57,78,93,109,137,138], though the therapeutic window has been extended in a few investigations to either 1 h [139,140] or 2 h post-injury [56]. In studies by our group, hypothermia was started at 5 min post-injury and maintained at 33 °C for 4 h [42]. In SCI work by Maybhate and colleagues [56], hypothermia was initiated approximately 2 h post-injury and maintained at 32 °C for 2 h. Both experimental studies showed beneficial histopathological effects and improved locomotor function, though the relationship between the timing of hypothermia post-SCI and its duration for therapeutic effect was not examined in either work through the use of manipulations of these parameters. It remains unclear in many SCI paradigms what time window for hypothermia after SCI will have the most beneficial effect, or what time window will be too late to have therapeutic effect.

In contrast to animal studies, the time window from the moment of SCI to the induction of hypothermia has often been much later in human, and the duration of hypothermia has been extended to 24 h and beyond (Table 1, [40,70,72,73,74,75]). In a recent clinical study, Dididze and colleagues [40] started modest systemic hypothermia (33 °C) within 5.76 ± 0.45 h of SCI, excluding cases in which the delay to treatment would be greater than 18 h. With this therapeutic window, evidence of a neurologic protective effect was observed when hypothermia was maintained for 48 h. This delayed initiation of hypothermia in clinical practice is often related to the patient’s time of transportation to clinical care as well as the time required to receive patients’ consent to accept hypothermia as an experimental treatment.

To date, however, this paradigm has not been investigated in experimental SCI and it remains unanswered whether the therapeutic window of hypothermia can be extended after SCI if the duration of hypothermia is prolonged. Previous use of hypothermia in TBI studies have suggested that cooling initiated beyond 8 h after trauma could be too late to still be effective [141]. In a rat cardiac arrest model, Che and colleagues [142] initiated therapeutic hypothermia (33 ± 1 °C) at 0, 1, 4, or 8 h after the recovery of spontaneous circulation and maintained it for 24 or 48 h. The seven-day survival rates were 45%, 36%, 36% and 14%, respectively, compared to 17% for the normothermia group, with no statistical difference between rats treated with 24 h therapeutic hypothermia and those treated with 48 h therapeutic hypothermia [142]. The survival with good neurologic outcome rates were 24%, 24%, 19% and 0%, respectively, compared to 2% for the normothermia group, and again with no statistical difference between rats treated with 24 h therapeutic hypothermia and those treated with 48 h therapeutic hypothermia [142]. Therefore, it appeared that no beneficial effects in the survival and neurological outcome was observed when hypothermia was initiated 8 h after the return of spontaneous circulation when it was maintained for up to 48 h [142].

**Table 1:** Initiation, duration and rewarming rate for systemic hypothermia in human SCI (2005–2015).

Year	Systemic Hypothermia	Initiation of Hypothermia	Duration of Hypothermia	Rewarming Rate
2015	-	-	-	-
2014	-	-	-	-
2013	Dididze *et al*., 2013 [40]	5.76 (±0.45) h from injury.	33 °C for 48 h	0.1 °C per hour until normothermia (T 37 °C)
2012	Madhavan *et al*., 2012 [74]	Immediately post surgery.	33 °C for 24 h	0.1 °C per hour until normothermia (T 37 °C)
2011	Tripathy and Whitehead, 2011 [75]	Case 1: The 3rd day post cervical SCICase 2: The 5th days of surgery after cervical SCI.	Case 1: a target temperature of 37.0–40.5 °C for 14 days Case 2: 37.0 °C for 10 days	Case 1: stopped at 40 °CCase 2: terminated on the 16th day of admission and gradually normalized by the 28th day
2010	Levi *et al*., 2010 [72]	The mean (standard error of the mean [SEM]) initiation time to catheter insertion was 9.17 (±2.24) h. If one excludes the 2 most aberrant outliers, the average time for initiation of hypothermia was 6.15 (±0.7) h.	33 °C for 48 h	0.1 °C per hour until normothermia (T 37 °C)
	Cappuccino *et al*., 2010 [70]	Temperature ranged between 34.5 and 35.2 °C with passive cooling during the surgery which was approximately 3 h after the cervical SCI, and then over 20 h post- injury lowed to 33.5 °C.	33.5 °C for 36 h	slowly rewarmed and eventually extubated on postoperative day 3
2009	Levi *et al*., 2009 [73]	The average time between injury and induction of hypothermia was 9.17 ± 2.24 h.	33 °C for 47.6 ± 3.1 h	0.1 °C per hour until normothermia (T 37 °C)
2007	-	-	-	-
2006	-	-	-	-
2005	-	-	-	-
2004	-	-	-	-

However, the initiation of hypothermia in SCI patients is more delayed practically, since it takes time to transfer and stabilize SCI patients before an intervention can be given, making it difficult to start hypothermia in the early hours post SCI. Therefore, it is necessary to investigate the efficacy of hypothermia with more extended therapeutic windows in experimental paradigms. A recent rat SCI study [143] tested the combination of delayed hypothermia (started 210 min after SCI, duration 3 h) with methylprednisolone (30 mg/kg, intravenously), given immediately after SCI. This combinational therapeutic paradigm showed much lower lipid peroxidation (MDA levels) than an early hypothermia application (started 30 min after SCI, duration of 3 h). This finding suggested that the immediate administration of the protective drug methylprednisolone has the potential to extend the therapeutic window of hypothermia.

### 6.2. The Duration of Hypothermia

The duration of hypothermia is another related parameter of hypothermia administration that is an important consideration for therapeutic effect. Currently, there has been no standard criteria to follow regarding the optimal period of hypothermia required for protection with either local cooling or systemic hypothermia administration and significant variation exists between experimental studies and human application.

In a dog thoracic spinal cord compression model, Wells and Hansebout [144] initiated local hypothermia (6 °C) at 4 h after compression injury and maintained it for 1, 4 or 18 h. The greatest degree of functional recovery was achieved in the 4 h duration group, rather than 1 or 18 h [145]. In experimental SCI studies using systemic hypothermia, the duration of hypothermia employed has been highly variable across published reports over the last decade (Table 2), being from minutes through to 48 h [41,42,55,56,57,78,93,109,132,137,139,141,145,146,147,148,149]. Recently, Vipin and colleagues [150] utilized uninjured rats to study the potential adverse effects of prolonged, semi-invasive, local hypothermia (30 ± 0.5 °C, durations of 5 or 8 h). No adverse effects were identified in the rats that underwent 5 or 8 h of hypothermia, with no statistical differences in measured somatosensory evoked potentials, histological parameters or BBB locomotor scores [150]. However, well-designed studies are still required to evaluate the most optimal hypothermia duration for maximal beneficial effect at clinically relevant therapeutic windows.

In human SCI studies, the duration of hypothermia has usually been at least 24 h (Table 1, [40,70,72,73,74,75]). As previously mentioned, in a sports-related cervical SCI case, Cappuccino and colleagues [70] maintained the patient at 33.5 °C for 36 h and they showed a rapid and significant neurological recovery from ASIA A to D; and Dididze and colleagues in a recent clinical investigation [40] initiated systemic modest hypothermia (33 °C) within 5.76 ± 0.45 h after SCI and maintained hypothermia for a duration of 48 h. In this study 43% (15 out of 35 patients) showed a recovery of at least one ISNCSCI grade.

**Table 2:** Initiation, duration and rewarming rate for systemic hypothermia in experimental SCI (2005–2015).

Year	Systemic Hypothermia	Initiation of Hypothermia	Duration	Rewarming Rate
2015	Wang and Zhang 2015 [133]	Probably immediately post-injury	34 ± 0.5 °C for 6 h	-
2014	Bazley *et al*., 2014 [140]	1 h post-injury and then 30 min induction phase	32 °C for 2 h	30 min to 37 °C
2013	Grulova *et al*., 2013 [41]	Immediately post-injury	31–32 °C was approximately 30 min	2 °C/h for 3 h to 37 °C
	Batchelor *et al*., 2013 [138]	82 min before the induction of injury to 30 min post-injury	28 to 34 °C from 31 min to 7.5 h	-
	Saito *et al*., 2013 [57]	Hypothermia was induced 15 min before ischemia	36.3 °C during ischemia (14 min)	rewarmed in 30 min
2012	Ok *et al*., 2012 [93]	Upon awaking from anesthesia	32 °C for 48 h	1 °C/h to normothermia
	Maybhate *et al*., 2012 [56]	Approximately 2.0 h post-injury	(32 ± 0.5 °C) for 2 h	28 ± 5 min to 37 ± 0.5 °C
2011	Batchelor *et al*., 2011, [55]	30 min following spacer insertion (spinal cord compression)	33 °C for 3.5 h	30 min to 37 °C
	Kao *et al*., 2011 [109]	From the compression termination period	33 °C for 2 h	Recover naturally
2009	Horiuchi *et al*., 2009 [147]	During ischemia	35 °C or 32 during ischemia	Rewarmed to 38 °C in 30 min
	Lo *et al*., 2009 [42]	5 min post-injury	33 °C for 4 h	1 °C per hour
	Duz *et al*., 2009 [146]	After spinal cord injury	27–29 °C for 1 h	Recover naturally
2008	Morino *et al*., 2008 [78]	The beginning of the compression	33 °C for 1 h	Recover naturally
2004	Tsutsumi *et al*., 2004 [141]	1 h after spinal cord ischemia-reperfusion	32.5 ± 0.5 °C for 6 h	1 h to normal temperature
	Strauch *et al*., 2004 [150]	Through the ischemia (clamping) period	32.0 °C through the ischemia (clamping) period	90–100 min
	Shibuya *et al*., 2004 [149]	At 10 min after the end of compression, lower body temperature to a target level over 20 min	32 °C for 4 h	40 min to 37 °C
2003	Maeda *et al*., 2003 [148]	During the ischemic period	35 °C for 30 min during the ischemic period	Gradually returned to 39 °C within 2 h

In TBI, Mclntyre and colleagues [151] examined therapeutic hypothermia of a duration of at least 24 h in a randomized control trial. They reported that compared with normothermia, 24 h of therapeutic hypothermia (32–33 °C) followed by rewarming over a subsequent 24 h, reduced the risk of a poor neurological outcome after TBI. Hypothermia longer than 48 h also reduced the risk of death. Che and colleagues [142] showed in a cardiac arrest model that the number of surviving neurons was greater when the duration of therapeutic hypothermia was 48 h compared to 24 h, suggesting that a prolonged therapeutic duration is associated with better neurological outcome. However, larger clinical investigations are needed to determine the most appropriate duration of systemic hypothermia that could be both safe and neuroprotective for acute SCI patients. Identifying whether longer durations of hypothermia can also achieve an extension in the therapeutic window for hypothermia administration would also be important to evaluate and allow its therapeutic use to be extended to a broader SCI population.

### 6.3. The Rate of Rewarming

**Figure 5:**
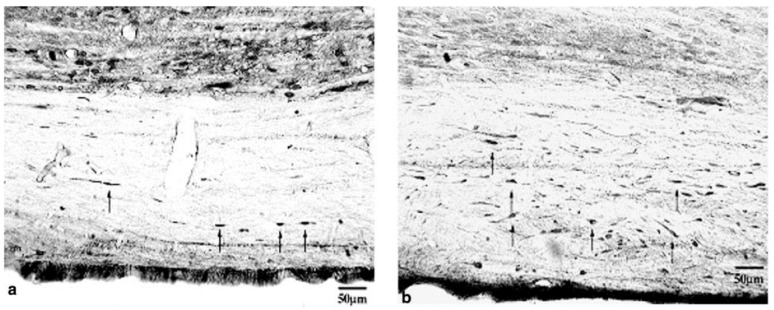
In these light microscopic images, we see the protective effects of hypothermic intervention followed by slow rewarming (**a**) *versus* the damaging effects associated with hypothermia followed by rapid rewarming (**b**) In both **a** and b, the damaged immunoreactive axons are labeled with arrows, with a striking demonstration of reduced axonal burden in **a** *versus* **b**. Reprinted with permission from [152], copyright Mary Ann Liebert, Inc., 2009.

**Figure 6:**
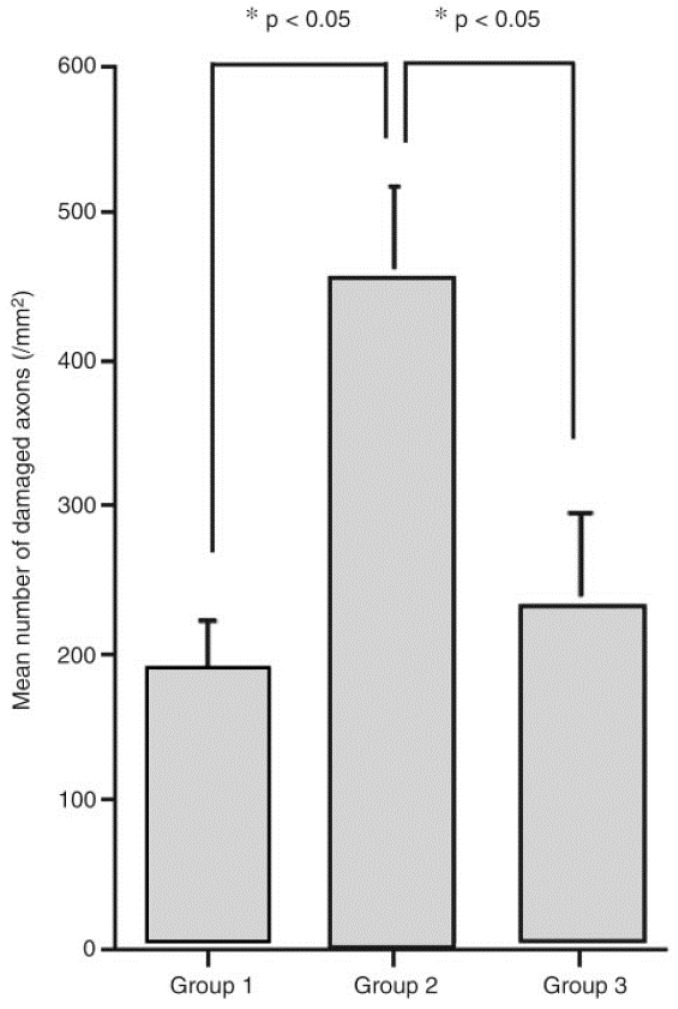
This bar graph shows a comparison of the numbers of amyloid precursor protein (APP) immunoreactive axonal profiles in the pontomedullary junction in three different treatment groups. Group 1 animals were subjected to traumatic brain injury (TBI) followed by 1 h of hypothermia and slow rewarming. In contrast, Group 2 animals were subjected to TBI and the same hypothermic intervention followed by rapid rewarming. Group 3 animals were also subjected to TBI followed by hypothermia and rapid rewarming with the concomitant infusion of cyclosporine A (CsA). Note that Group 1 animals showed a reduced burden of axonal damage associated with the use of hypothermia and slow rewarming, whereas these axonal numbers were dramatically increased following the same insult and hypothermic intervention, now with the inclusion of a rapid rewarming rate. Lastly, Group 3 was treated in the same fashion as Group 2, with the exception that CsA was administered prior to the initiation of rapid rewarming. Collectively, this figure illustrates the damaging effects of rapid posthypothermic rewarming and its attenuation via the use of the immunophilin ligand CsA. Reprinted with permission from [152], copyright Mary Ann Liebert, Inc., 2009.

In addition to the time window to initiate hypothermia and its duration, another important topic that deserves consideration is the rate of rewarming from hypothermia back to normothermia. Previous work in TBI has demonstrated that greater neuroprotection was achieved with slower rewarming, in contrast to rapid rewarming, which resulted in a deleterious effect [152,153]. In a rat weight drop TBI model, Suehiro and Povishock [153] compared the effects of slow rewarming with fast rewarming on axonal injury. Systemic hypothermia (32 °C) was initiated immediately after TBI and maintained for a 1 h duration. Animals were then allocated to either a slow rewarming group (90 min, group 1) or fast rewarming group (20 min, group 2), or fast rewarming (20 min) with the intrathecal cyclosporine A (CsA) infusion before rewarming (group 3). CsA is an immunophilin ligand that possesses the ability to provide mitochondrial protection by inhibiting the permeability transition pore on the inner mitochondrial membrane. At 24 h post-injury, less axonal damage, as visualized by fewer APP-immunoreactive axonal profiles, was identified within the corticospinal tract in the slow rewarming group (group 1) compared to the fast rewarming group (group 2); exacerbated axonal damage by fast rewarming (group 2) could be significantly reduced, however, by the use of CsA (group 3) (Figure 5 and Figure 6, respectively) [152]. Additionally, Suehiro and colleagues [154] demonstrated that slow rewarming after therapeutic hypothermia provided better protection of cerebral microcirculation and maintained normal arteriolar vascular responses. In contrast, rapid rewarming (within 30 min), impaired cerebral vascular responses to acetylcholine and arterial hypercapnia [155]. Similarly, in a recent human TBI study, it was shown that a rapid rewarming rate (faster than 0.25 °C/h) was associated with worse outcomes as demonstrated by a higher mortality rate, longer length of stay in the intensive care unit (ICU) and lower Glasgow Coma Scale (GCS) scores at hospital discharge [156].

In the field of SCI, however, there are no standards to follow in terms of the most optimal rewarming rate to employ after hypothermia administration. While some studies have used natural rewarming at room temperature or on a heating pad with no temperature records on timed recovery (Table 1, [78,109,145]), others have rewarmed the animals for a period of about 30 min (Table 1, [55,56,57]). There have also been reports of more standardized protocols involving rewarming animals at a rate of 1 °C/h [42,93] or 2 °C/h [41]. In a rat cervical SCI study from our group, Lo and colleagues [42] rewarmed animals at 1 °C per hour over a period of approximately 4 h back to normothermia, a slow rewarming paradigm. However, there have been no reports to compare the effect of fast rewarming and slow rewarming in SCI animal experiments. In human SCI, Dididze and colleagues [40] carried out rewarming even more slowly, at 0.1 °C/h for SCI patients, taking about 40 h to rewarm an individual from 33 to 37 °C. In light of the results from TBI studies that demonstrated improved axonal protective effects and the maintenance of the vascular responses by slow rewarming [152,153,154], it is possible that slow rewarming will be more beneficial than rapid rewarming after SCI. Further work is required to investigate the optimal rewarming rate and its standardization for human SCI application.

Another crucial consideration is that rewarming should not exceed 37 °C. In a human TBI study, Lavinio and colleagues [157] reported that after moderate hypothermia (34.2 °C), rewarming above the brain temperature threshold of 37 °C was associated with an increase in the average cerebrovascular pressure reactivity index (PRx), indicating a significant derangement of cerebral autoregulation. Further, a SCI study in rats has demonstrated that systemic hyperthermia itself worsened locomotor outcome and increased tissue damage [158]. Therefore, rewarming durations and temperature limits are important considerations for recovery after hypothermia application in SCI.

## 7. Conclusions

In summary, hypothermia is a promising neuroprotective treatment for SCI. There is a strong potential to combine hypothermia with other therapeutic approaches, such as pharmaceuticals and cellular transplantation for more robust effects on histological and functional outcomes. Particularly, it is beneficial to investigate the potential synergistic neuroprotective effect for SCI through the combination of hypothermia with erythropoietin and derivatives, riluzole (a sodium channel-blocking benzothiazole anticonvulsant medication), calpain inhibitors (e.g., AK295, MDL28170, SJA6017), minocycline, Rho kinase inhibitors (Fasudil) or Schwann cell transplantation. Further, large multicenter trials are required to investigate a standardized and optimized protocol to achieve the most beneficial effect of clinical hypothermia therapy for SCI. Specifically, the therapeutic window, approaches to extend this window, the optimal duration of hypothermia as well as the standardization of the rewarming protocol are all at the forefront of the current research gap.
